# Gold Nanoparticle-Mediated Delivery of Molecules into Primary Human Gingival Fibroblasts Using ns-Laser Pulses: A Pilot Study

**DOI:** 10.3390/ma9050397

**Published:** 2016-05-20

**Authors:** Judith Krawinkel, Maria Leilani Torres-Mapa, Kristian Werelius, Alexander Heisterkamp, Stefan Rüttermann, Georgios E. Romanos, Susanne Gerhardt-Szép

**Affiliations:** 1 Institute of Applied Optics, Friedrich-Schiller-University Jena, Fröbelstieg 1, Jena 07743, Germany; judith.krawinkel@uni-jena.de; 2 Institute of Quantum Optics, Gottfried Wilhelm Leibniz University Hannover, Welfengarten 1, Hannover 30167, Germany; torres@iqo.uni-hannover.de (M.L.T.-M.); heisterkamp@iqo.uni-hannover.de (A.H.); 3 Department of Postgraduate Education, J.W. Goethe University, Theodor-Stern-Kai 7, Frankfurt am Main 60590, Germany; werelius@em.uni-frankfurt.de; 4 Department of Operative Dentistry, Carolinum Dental University-Institute GmbH, J.W. Goethe University, Theodor-Stern-Kai 7, Frankfurt am Main 60590, Germany; ruettermann@med.uni-frankfurt.de; 5 Department of Periodontology, School of Dental Medicine, Stony Brook University, Stony Brook, NY 11794, USA; georgios.romanos@stonybrook.edu; 6 Department of Oral Surgery and Implant Dentistry, Carolinum Dental University-Institute GmbH, J.W. Goethe University, Theodor-Stern-Kai 7, Frankfurt am Main 60590, Germany

**Keywords:** laser based cell manipulation, human gingival fibroblasts, gold nanoparticles, laser

## Abstract

Interaction of gold nanoparticles (AuNPs) in the vicinity of cells’ membrane with a pulsed laser (λ = 532 nm, τ = 1 ns) leads to perforation of the cell membrane, thereby allowing extracellular molecules to diffuse into the cell. The objective of this study was to develop an experimental setting to deliver molecules into primary human gingival fibroblasts (pHFIB-G) by using ns-laser pulses interacting with AuNPs (study group). To compare the parameters required for manipulation of pHFIB-G with those needed for cell lines, a canine pleomorphic adenoma cell line (ZMTH3) was used (control group). Non-laser-treated cells incubated with AuNPs and the delivery molecules served as negative control. Laser irradiation (up to 35 mJ/cm^2^) resulted in a significant proportion of manipulated fibroblasts (up to 85%, compared to non-irradiated cells: *p* < 0.05), while cell viability (97%) was not reduced significantly. pHFIB-G were perforated as efficiently as ZMTH3. No significant decrease of metabolic cell activity was observed up to 72 h after laser treatment. The fibroblasts took up dextrans with molecular weights up to 500 kDa. Interaction of AuNPs and a pulsed laser beam yields a spatially selective technique for manipulation of even primary cells such as pHFIB-G in high throughput.

## 1. Introduction

Molecular delivery methods are key technologies in the biomedical sciences. Despite several physical methodical approaches (ballistic, electricity, iontophoresis, ultrasound, light) the efficient delivery of molecules to cells remains challenging [1,2]. Schneckenburger *et al.* introduced absorption of laser energy (2.5 MJ/cm^2^ for 1 s) by phenol red to transfect cells [3]. In order to apply lower radiant exposures (RE) absorbing nanoparticles were utilized to induce plasmonic effects. Short laser pulses interact with nanoparticles leading to localized, transient increases of cell permeability without affecting cell viability [2,4]. Lasers interacting with nanoparticles were shown to be able to efficiently deliver molecules into cells [2,4,5]. Jumelle *et al.* delivered calcein molecules into corneal endothelial cells by carbon nanoparticles activated by a femtosecond laser. The uptake reached median efficiency of 54.5% with low (0.5%) mortality [2]. St-Louis Lalonde *et al.* compared membrane permeabilization by irradiating AuNPs with ns-laser pulses on- (532 nm) and off- (1064 nm) resonance [5]. Another transfection technique described in literature is laser scanning of cells previously incubated with gold nanoparticles (AuNPs), called the GNOME approach. Applying the GNOME technique, Heinemann *et al.* already described the possibility to deliver green fluorescent proteins into mammalian cells with an efficiency of 43%, while maintaining a high level of cell viability. Compared to conventional transfection techniques the GNOME method enables high-throughput transfection of about 10,000 cells per second [1]. Additionally the cell survival rate is high because the effects of this method are highly localized [1]. Depending on the experimental objectives, the laser parameters can be modified to not only achieve reversible cell perforation but even induce targeted cell apoptosis [1]. Lukianova-Hleb *et al.* utilized plasmonic nanobubbles generated upon laser irradiation of AuNPs to mechanically eliminate cells and tissue, proposing their method as a precise micro-surgical tool [6]. Besides nanobubbles, laser induced shock-waves were also utilized to deliberately damage cell membranes [7], deliver photosensitizers into biofilms for their eradication [8], or to transfect cells *in vivo* and *in vitro* [9].

Incubation of cells with AuNPs leads to the attachment of the particles to the cell membrane. Laser irradiation results in plasmonic effects on the AuNPs, field enhancement around the particles, and increased local heat [10,11,12,13,14,15]. Utilizing these effects, large cell areas can be irradiated quickly while avoiding the need to laser irradiate individual single cells. If appropriate RE (energy received per surface area) is applied, transient membrane perforation may result in areas where AuNPs are adjacent to the cell membrane [10,16]. Non-irradiated cells or cells without AuNPs attached [1] are not damaged by laser irradiation at the chosen RE. Thus, the method is suitable for selective manipulation of cells, both in temporal and spatial terms, because the timing as well as the area of irradiation can be selected individually. Available studies on the laser parameters reported in the literature employed cell lines rather than primary cells [1], or involved an fs-laser (λ = 780 nm) [17]. For the latter, the optimal RE found for a carcinoma cell line was directly transferred to primary cells resulting in a transfection efficiency of 2.7% and cell losses of around 65% [17]. For these cells the optimal RE has not been studied. In the present article, we describe for the first time the delivery of different molecules into primary human gingival fibroblasts (pHFIB-G) using AuNPs and laser irradiation. There is no information in the literature with regard to the REs that are associated with the highest number of perforated primary HFIB-G while maintaining cell viability. We assume a different reaction of primary human cells compared to those of a rather robust carcinoma cell line when being exposed to the interaction of AuNPs and laser pulses. This would indicate the necessity to carefully study possible negative side effects on the pHFIB-G and how to minimize them in order to reasonably transfer the results published earlier on this technique to clinical settings.

Our study hereby closes the important gap in applying this method in human cells and compares the findings in primary cells with those reported for cell lines. Thus, our two research questions were the following:

1. Can pHFIB-G be efficiently manipulated by ns-laser pulses interacting with AuNPs while maintaining high cell viability in comparison to a cell line (ZMTH3)?
2. Does laser irradiation allow spatial selectivity of treated cells, and can molecules be incorporated into pHFIB-G?

## 2. Materials and Methods

### 2.1. Materials and Cells

We used primary human gingival fibroblast cells (HFIB-G, provitro GmbH, Berlin, Germany) cultured in Dulbecco’s Modified Eagle Medium (DMEM) with 10% fetal calf serum (FCS) and 1% penicillin/streptomycin (all obtained from Biochrom GmbH, Berlin, Germany). The cells were incubated at 37 °C and 5% CO_2_. Depending on the experiments, they were either seeded in 35 mm glass-bottom dishes or in 24- or 96-well plates (glass bottom) one day before the experimental procedure.

To compare the laser parameters required for manipulation of pHFIB-G with those needed for cell lines, a canine pleomorphic adenoma cell line (ZMTH3) [18], provided from the Small Animal Clinic, University of Veterinary Medicine Hannover, Germany was used. ZMTH3 were cultured in Roswell Park Memorial Institute medium (RPMI-1640) with 10% FCS and 1% penicillin/streptomycin. Incubation, cell preparation, and laser treatment were the same for all experiments. The temperature was kept at 37 °C throughout the experiment.

### 2.2. Nanoparticles and Cell Manipulation

We used 200 nm AuNPs (Kisker Biotech, Steinfurt, Germany) at a concentration of 0.5 μg/cm^2^. The cells were incubated with AuNPs for 3 h. Within this time the particles sedimented on the cell surface. After incubation, the cell medium containing AuNPs was removed, retaining the sedimented particles on the cells. It was replaced by fresh DMEM containing the molecules to be delivered and 2% HEPES buffer solution (1 M, Sigma Aldrich, St. Louis, MO, USA) to maintain a physiological pH level during laser treatment. Then, the cells were irradiated with a laser.

### 2.3. Laser Irradiation and Assessment of Molecules Uptake

**Figure 1:**
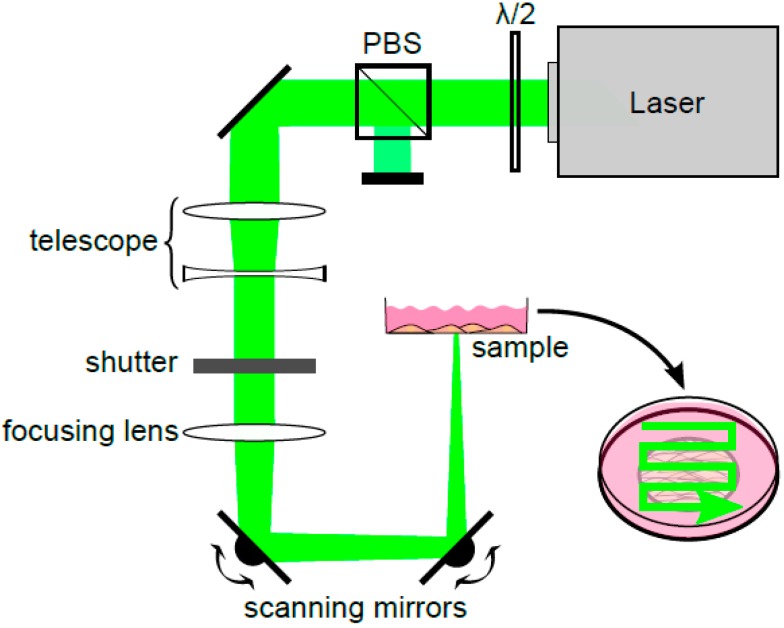
Schematic representation of the irradiation setup. For power adjustment, the combination of a half-wave plate (λ/2) and a polarizing beam splitter cube (PBS) was used. The laser beam was collimated with the help of a telescope and focused with a lens (f = 250 mm). To treat all cells in the region of interest, scanning mirrors allowed the laser beam to raster scan the entire sample as shown in the inset on the lower right.

To perforate all cells within a short time, a pulsed HLX-G-F020 microchip laser (Horus Laser, Limoges, France) emitting at a wavelength of 532 nm was applied to the entire sample area by raster scanning the beam. Laser pulse duration was 1 ns and the repetition rate was 22.5 kHz. In the setup (Figure 1) the laser output power was adjusted using a half-wave plate (λ/2) and a polarizing beam splitter cube (PBS).

A telescope collimated the laser beam. To focus the beam, a lens (focal length f = 250 mm) was used resulting in a focus diameter of the beam of 100 μm and entailing a large Rayleigh range of about 16.2 mm. Hence, the REs in the whole sample region were assumed to be constant despite scanning the beam using two scanning mirrors (*x*- and *y*-direction). With this relatively weak focusing REs up to 35 mJ/cm^2^ were obtained. Furthermore, high throughput was possible because irradiation of a cell growth area of 1 cm^2^ took approx. 60 s when scanning with a speed of 50 mm/s and a lateral distance between two scanning lines of 33 μm.

After laser irradiation the cells were incubated for 10 min before washing them twice with phosphate-buffered saline. Next, fresh medium was added to allow continued culturing of cells or to determine the yield as described below. For quantification purposes each sample was handled in a separate well and all cells within one well were treated. When irradiating a user defined pattern of cells no quantification was performed.

To define the optimal laser parameters for the manipulation technique, fluorescent dyes were used. First, perforation efficiency *(i.e.*, the number of cells successfully perforated but still viable) was determined. Therefore, 0.5 mM of the cell-impermeable, fluorescent dye calcein (Sigma Aldrich, St. Louis, MO, USA) was dissolved in the extracellular medium before irradiation. It only enters the cytoplasm of those cells that were perforated during laser irradiation. After removing the extracellular calcein molecules, the successfully perforated cells fluoresced. Perforation efficiency was determined by counting the proportion of calcein-fluorescing cells. Simultaneously, the number of necrotic cells was determined. For quantification, the cells were first detached using TrypLE (Life Technologies, Carlsbad, CA, USA) and the TrypLE reaction was stopped by adding cell culture medium. Afterwards, the cells were centrifuged (3 min at 300× *g*), the supernatant was removed, and the cells were resuspended in 20 μL of medium. Then, 2.5 μM of the fluorescent dye propidium iodide (PI, Life Technologies) was added to the cell suspension to stain necrotic cells. A Cellometer Vision 5x (Nexcelom Bioscience, Lawrence, MA, USA) was used to determine the total number of cells as well as the number of calcein- and PI-positive cells. For each parameter the experiment was performed on three different days.

For the experiment, pHFIB-G as well as the cell line ZMTH3 were irradiated with REs up to 35 mJ/cm^2^. Non-laser-treated cells incubated with AuNPs and the delivery molecules served as negative control. When determining the proportion of perforated cells, only viable cells were counted. Perforation efficiency was defined as the percentage of cells containing calcein but no PI of the total number of treated cells. Thus, successfully perforated cells were those that were transiently permeabilized but were still able to seal their membrane afterwards so that no PI could enter. Non-necrotic cells were those that were successfully perforated but did not contain any PI. Long-term viability and cell proliferation were determined separately. To test whether larger molecules were delivered into the cells, fluorescein isothiocynate (FITC)-labeled dextrans of different molecular weights (10 kDa, 70 kDa, and 500 kDa) were dissolved in the cell culture medium. Preparation, laser irradiation, and washing steps were the same as those described above.

### 2.4. Viability Assay

Cell viability was determined by counting non-necrotic cells immediately after every experiment. Long-term influence of the laser treatment on the cells was determined by a proliferation assay using PrestoBlue (Life Technologies). The cells were seeded in a 96-well plate with 1.5 × 10^4^ cells/well. Afterwards, cells were treated with AuNPs and laser as described above. To measure PrestoBlue reduction, the cell culture medium was removed from the wells and was replaced by fresh medium containing 10% PrestoBlue. Next, the cells were incubated at 37 °C and 5% CO_2_ for 24 h. Forty-eight hours, and 72 h after laser treatment absorbance of PrestoBlue at 570 nm was measured using a SPECTROstar Omega (BMG LABTECH, Ortenberg, Germany) plate reader. Emission at 600 nm was measured as a reference wavelength to normalize the absorbance. All values were background-corrected by subtracting the value for PrestoBlue reduction in medium alone. PrestoBlue reduction in non-treated cells (no AuNPs, no laser irradiation) was used as positive control. Two additional control groups were applied to identify the influence of laser irradiation and AuNPs separately: pHFIB-G treated with AuNPs but without laser irradiation, and cells treated without AuNPs, but irradiated with the highest RE. Cells were killed by incubating them in a 1:1 solution of methanol (99.6%) and ethanol (99%) for 10 min served as negative control. All parameters were analyzed in triplicates (*n* = 3) with each 1.5 × 10^4^ cells per parameter.

To determine perforation specificity and efficiency for different molecules, the cells were treated as described above. To image the fluorescent cells an inverted microscope (Axiovert A.1, Zeiss, Oberkochen, Germany) and an EMCCD-Camera (iXon DU-885, Andor Technology Ltd., Belfast, UK) using the Andor Solis image acquisition software were used. All images were post-processed using ImageJ (U.S. National Institutes of Health, Bethesda, MD, USA) by enhancing their intensity and contrast as well as false color representation.

### 2.5. Statistical Analysis

The impact of laser irradiation on perforation efficiency and percentage of non-necrotic cells were analyzed in cooperation with the Institute of Biostatistics and Mathematical Modelling for different REs for pHFIB-G and ZMTH3 by using a non-parametric one-way analysis of variances (ANOVA) using ranks for non-normally distributed data.

## 3. Results

**Figure 2:**
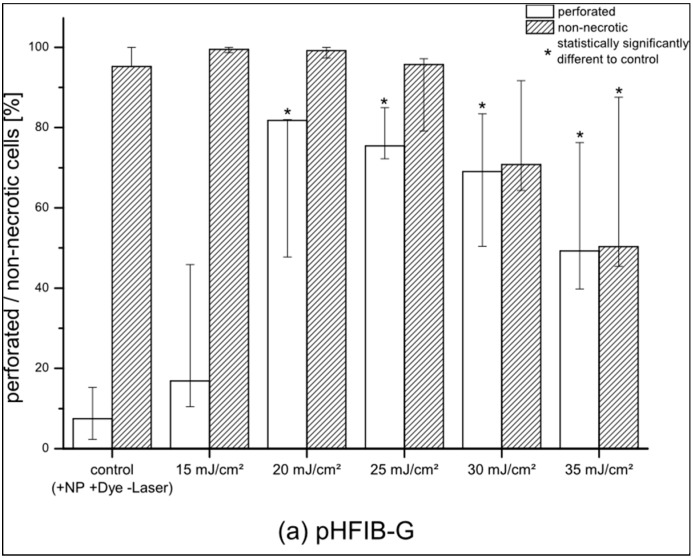

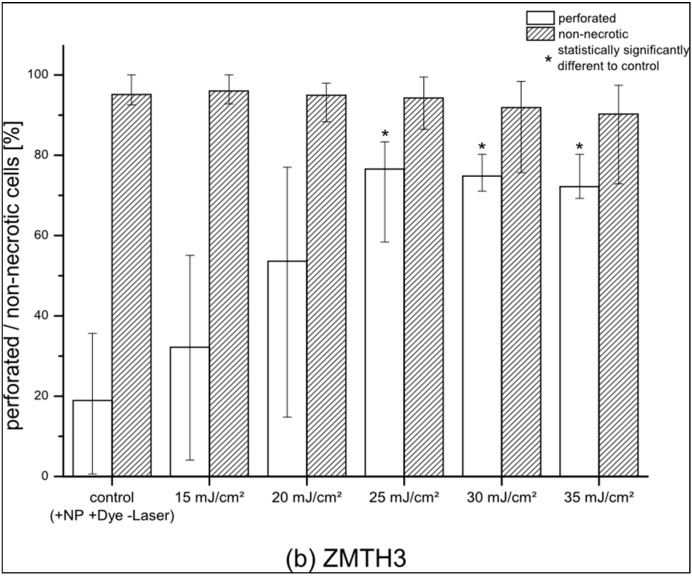
Perforation efficiency and percentage of non-necrotic cells irradiated with different radiant exposures (REs) for (**a**) pHFIB-G and (**b**) cell line ZMTH3 (statistical significance: *p* < 0.05). For all parameters the median as well as minimum and maximum percentages are shown (*n* = 3).

**Figure 3:**
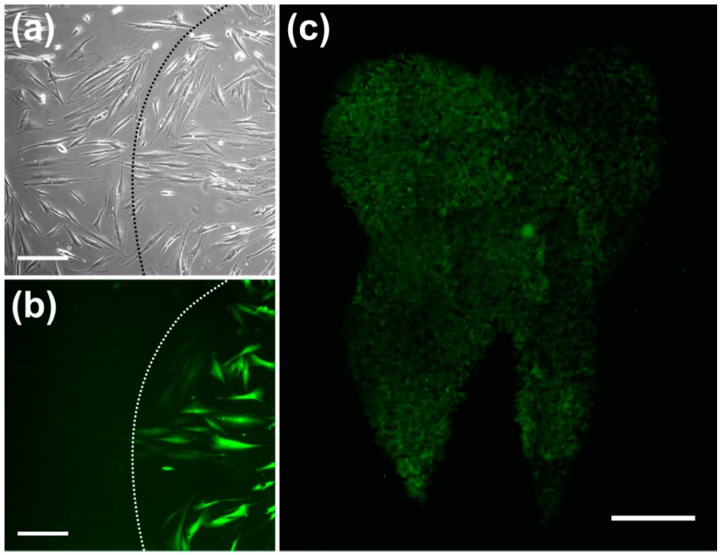
The technique allows spatially-specific irradiation. Only those pHFIB-G that were irradiated with a laser at a RE of 25 mJ/cm^2^ were perforated and took up calcein (**b**, right side of dashed line); Bright-Field image of the same region (**a**) reveals that cells are evenly distributed and no differences between irradiated and non-irradiated area are visible. Still, only the pHFIB-G in the irradiated area were perforated (**b**); The irradiated cell area is a user defined pattern (e.g., in the shape of a tooth) (**c**). Scale bars: (**a**,**b**) 200 μm; (**c**): 2000 μm.

pHFIB-G and ZMTH3 were irradiated with REs up to 35 mJ/cm^2^. The proportion of perforated living pHFIB-G increased with REs up to 20 mJ/cm^2^ and decreased for higher REs (Figure 2a). At REs between 20 mJ/cm^2^ and 35 mJ/cm^2^ the difference between the control group of non-irradiated cells or cells irradiated with lower REs (e.g., 15 mJ/cm^2^) was statistically significant (*p* < 0.05). Up to REs of 20–25 mJ/cm^2^ the percentage of cells that successfully took up calcein increased up to a maximum of 85%, while 97% of these cells were non-necrotic. As perforation efficiency was based on the proportion of living cells, it decreased in parallel with decreasing cell viability. Hence, REs of 20 to 25 mJ/cm^2^ achieved highest perforation efficiency with high cell viability (Figure 2a). Comparing the results obtained for pHFIB-G with those obtained for a cell line (ZMTH3), we found that—in contrast to the finding for pHFIB-G—the proportion of necrotic ZMTH3 cells did not increase significantly at REs up to 35 mJ/cm^2^ (Figure 2b). We ascribe the comparably high amount of calcein positive ZMTH3 without laser irradiation to the higher endocytic activity of tumor cells [19] during which they naturally take up molecules. Irradiating ZMTH3 with REs of 25 mJ/cm² and higher leads to a statistically significant (*p* < 0.05) increase in calcein uptake compared to non-irradiated cells. A maximum of 83% of ZMTH3 cells were perforated when the laser irradiated at a RE of 25 mJ/cm^2^ while more than 99% of the cells were viable. Hence, 25 mJ/cm^2^ was chosen as the optimal RE (Figure 2). Using this RE, pHFIB-G in a spatially defined cell growth area in shape of a tooth (Figure 3) were partly irradiated. Even though all of the pHFIB-G were incubated with AuNPs, only laser irradiated cells were perforated (Figure 3b,c). A magnified view of the boundary of such an irradiation pattern (Figure 3b) reveals no differences between the irradiated and non-irradiated area in bright-field images (Figure 3a).

Through the small membrane openings, extracellular medium diffused into the cells due to concentration differences which, therefore, took up the extracellular fluorescent dye. A bright-field microscopic image (see Figure 3a) revealed no obvious difference between irradiated and non-irradiated cells. However, fluorescence microscopic analysis of the same area showed that only a part of the cells was perforated (Figure 3b). Due to the relatively weak laser focusing applied a high throughput was achieved. The irradiation of an area of 1 cm^2^ took about one minute (Figure 3c).

**Figure 4:**
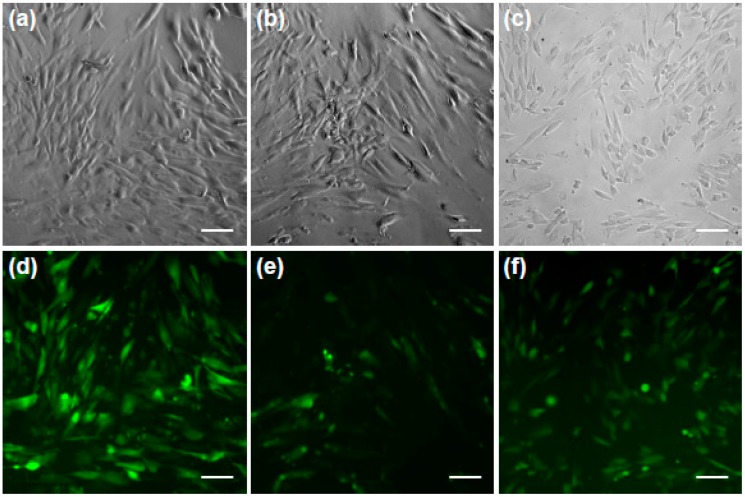
pHFIB-G irradiated with a RE of 25 mJ/cm^2^ can also take up molecules larger than calcein. pHFIB-G took up fluorescently labeled dextrans with molecular weights (**d**) 10 kDa; (**e**) 70 kDa; and (**f**) 500 kDa dextrans; as shown in the bright-field images (**a**–**c**); All scale bars: 35 μm.

Microscopic images confirm that fluorescently labeled dextrans with molecular weights of 10 kDa, 70 kDa, and 500 kDa reached the cytoplasm if the optimal RE of 25 mJ/cm^2^ was applied (see Figure 4; bright-field images a–c and the according fluorescent images d–f).

**Figure 5:**
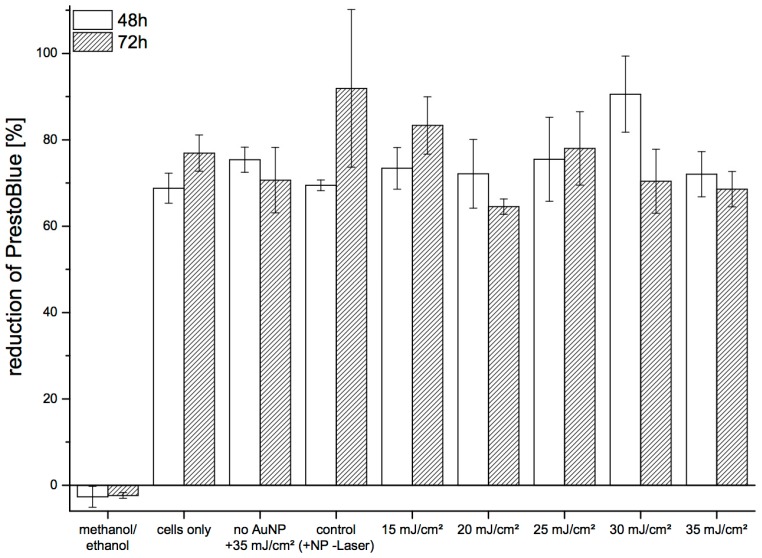
Long-term influence of the experimental procedure on metabolic activity of pHFIB-G measured by the reduction of PrestoBlue 48 h and 72 h after laser irradiation. All values are background-corrected for the blank (cell culture medium only). Means and SE are shown (*n* = 3).

We assessed long-term viability of pHFIB-G based on the reduction of Presto-Blue in the cell culture medium (Figure 5). Metabolic activity of the cells was determined at 48 h and 72 h after laser irradiation. No significant decrease of metabolic cell activity was observed up to 72 h after laser treatment, with the reduction of PrestoBlue at 48 h and 72 h found to be comparable. No trend of decreasing cell viability was seen when irradiating the cells with increasing REs. Similarly, the level of reduction for treated cells was in the same range as for non-treated cells. Reduction of PrestoBlue was 69%–92% in all samples except the negative control (cells killed with methanol/ethanol) (Figure 5).

## 4. Discussion

In the literature, the GNOME method used here is described as highly efficient and gentle to cells, and represents a promising tool in the search of medical applications of molecules [10]. Recent studies using on- and off-resonant laser pulses of different wavelengths systematically assessed the parameters that influence perforation rate and cell viability [2,5,10,20]. In contrast to continuous-wave laser exposure [21], pulsed laser irradiation as used in our experiments (interval between laser pulses: 44.4 μs) does not lead to accumulation of thermal effects because AuNPs are able to conduct the heat to their environment within nanoseconds [22]. Furthermore, conjugation of AuNPs with specific cell markers to further improve the selectivity is feasible. However, excessively high REs may induce irreversible damage to the cell membrane that cannot be repaired by the cell. This leads to necrosis and apoptosis of the targeted cells [12,15,23,24,25,26].

Successful transfection of cells with fluorescent siRNA as well as knockdown of the oncogene HMGA2 in tumor cells with specific siRNAs were demonstrated elsewhere [20]. Furthermore, the passive binding of AuNPs to the cell membrane has already been studied as well [10,20]. siRNA transfection models investigating future therapeutic strategies in dentistry were also recently investigated [26]. Authors found that p63 overexpression, for example, was associated with increased radioresistance and an unfavorable outcome for oral cancer patients [27]. Mueller *et al.* showed already that primary fibroblasts could be manipulated through nucleofection for transplantation purposes. These fibroblasts were treated with an efficiency of about 63% and transgene expression showed persistence up to day nine post-nucleofection [28]. The utilization of primary gingival fibroblast cells has many additional advantages: they are readily available in patients and thus represent a promising platform for cell-based therapies. However, no corresponding studies exist with these special gingival cells.

Perforation parameters for the cell line ZMTH3 using the GNOME method were, however, studied previously, and these were applied to different cell types without examining their optimal RE systematically [10,20,29]. We applied this technique on primary human gingival fibroblasts for the first time and studied the perforation parameters of primary human cells compared to those required for a cell line as we assume them to respond differently to the occurring side effects. The stated optimal RE (25 mJ/cm^2^) to perforate pHFIB-G coincided with the optimal RE for the cell line ZMTH3. This is in good accordance with the optimal RE of 20 mJ/cm^2^ determined by Heinemann *et al.*, who used a laser with pulse duration of 0.85 ns [10]. St-Louis Lalonde *et al.* found an optimal RE 55 mJ/cm^2^ when applying 15 ns laser pulses [5]. Even though pHFIB-G seemed to be more sensitive than ZMTH3 cells, and their viability was reduced when using higher REs, perforation efficiencies with the same experimental conditions reached similar maximum values (85% and 83%, respectively). We ascribe the reduced viability for pHFIB-G to generation of ROS (Reactive Oxygen Species) during laser irradiation which can lead to cell death [24]. This effect is more pronounced in pHFIB-G as cancer cells are capable of dealing with higher ROS levels due to their higher antioxidant capacity [30]. Further optimization remains possible to increase the RE and the efficiency parameters. Solutions also exist to decrease the mortality triggered by molecule delivery. In this context recently synthesized and modified anti-oxidant molecules with ascorbic acid (PPAA) at various ratios showed collagen synthesis promoting property [31]. Weibel *et al.* investigated the laser-assisted delivery of Vitamin C, E, and Ferulic to achieve greater penetration in the tissue and stated an increased beta fibroblast growth factor expression [32]. Güney *et al.* succeeded to incorporate ascorbic acid (Vitamin C) in solid lipid nanoparticles (SLNs) by hot homogenization and to deliver them efficiently to cancer cells [33]. Our next step is therefore to assess the extent of which ascorbic acid could reduce cell death during molecule delivery by ns-laser pulses, while retaining high efficiency. These questions are also processed by different author teams, working on comparable models [2]. However, it must be stated, that *in vitro* experiments cannot be directly extrapolated to the intact *in vivo* situation, although we used the human cells most similar to the native tissue. The use of primary cells however presents huge donor to donor variability in terms of population doubling time and of morphology [2].

Hence, the technique seems not to be dependent on the cell type and can be used in cell lines as well as in primary human cells, even though they show different sensitivity towards the induced side effects. Spatial selectivity of the method was shown using the membrane impermeable dye calcein as a fluorescent marker. Only in irradiated regions did the absorption of laser energy by the AuNPs lead to membrane perforation. Absorption by phenol red as utilized in [3] can be neglected due to 10^5^ times smaller REs applied and lower absorption of phenol red at 532 nm compared to 488 nm. Additionally, for ZMTH3 irradiation of cells without AuNPs were shown not to perforate the cellular membrane [1]. As analogously no effect was expected for pHFIB-G this was not examined in the present study.

Non-treated cells (no AuNPs, no laser irradiation) and cells not incubated with AuNPs but irradiated with laser at a RE of 35 mJ/cm^2^ showed the same level of PrestoBlue reduction as did irradiated cells. Neither laser-irradiation nor AuNP-incubation or their interaction led to a decrease in cell proliferation and, consequently, long-term viability of pHFIB-G. Our findings prove that only irradiated cells were transiently perforated.

Heinemann *et al.* also showed that the absorbance spectra change with higher REs as AuNPs may melt, although the temperature induced by the RE applied are below the melting point of gold (~1063 °C) [10,15]. They proposed that thermal effects accompanied by multiphoton effects may be responsible for membrane perforation.

After an incubation time of 3 h, the AuNPs applied sedimented and adhered to the cell surface, resulting in about six particles per cell for a AuNP concentration of 0.5 μg/cm^2^ [10]. Compared to off-resonance (796 nm) laser-mediated cell manipulation, this number of particles is low [20]. As no particle accumulation is necessary, defined perforation of the cell membrane in the vicinity of AuNPs is possible. Applying AuNPs conjugated to antibodies [24] or other cell-type specific markers may allow secondary spatial selection as only specific cells are targeted. Furthermore, we demonstrated that GNOME-treated pHFIB-G are also able to take up molecules at a size of up to 500 kDa. Hence, the results of delivering proteins and nucleic acids reported by Schomaker *et al.* [29] and Heinemann *et al.* [1,10] should be transferable to pHFIB-G using the same parameters. By utilizing media additives like antioxidants to reduce negative side effects of GNOME, it is likely to increase the viability and, hence, the transfection efficiency of primary cells. With this, the optimal RE for cells lines can be transferred to primary cells. This is promising for future applications in cellular therapies as the accessibility of gingival fibroblasts is high.

## 5. Conclusions

This study demonstrates that gold nanoparticle-mediated manipulation of primary human gingival fibroblasts (pHFIB-G) using ns-laser pulses is a highly efficient method in cell perforation and is associated with low cytotoxicity. The best efficiency/toxicity ratio was obtained at a RE of 25 mJ/cm^2^ with 85% efficiency and toxicity below 3%.

It also anticipates what could be done on pHFIB-G to improve outcomes in future cell or tissue therapy.

## Abbreviations

The following abbreviations are used in this manuscript:

AuNP	Gold nanoparticles
RE	Radiant exposure
pHFIB-G	Primary human gingival fibroblasts
ANOVA	Non-parametric one-way analysis of variances
ZMTH3	Canine pleomorphic adenoma cell
GNOME	Laser-based gold-nanoparticle mediated manipulation of cells
FCS	Fetal calf serum
DMEM	Dulbecco’s Modified Eagle Medium
RPMI-1640	Roswell Park Memorial Institute Medium
PBS	Polarizing beam splitter cube
PI	Propidium Iodide
FITC	Fluorescein isothiocynate
